# Alcohol consumption is associated with the risk of developing colorectal neoplasia: Propensity score matching analysis

**DOI:** 10.1038/s41598-019-44719-w

**Published:** 2019-06-04

**Authors:** Young Joo Yang, Chang Seok Bang, Jae Ho Choi, Jae Jun Lee, Suk Pyo Shin, Ki Tae Suk, Gwang Ho Baik, Dong Joon Kim

**Affiliations:** 1 Department of Internal Medicine, Hallym University College of Medicine, Chuncheon, Korea; 2 Institue of New Frontier Research, Hallym University College of Medicine, Chuncheon, Korea; 3 Department of Anesthesiology and Pain Medicine, Hallym University College of Medicine, Chuncheon, Korea

**Keywords:** Colonoscopy, Colon cancer

## Abstract

Although alcohol intake is known to be associated with the development of colorectal cancer, the effect of alcohol consumption on the development of colorectal neoplasm (CRN) is unclear. We performed a retrospective cohort analysis with 1 to 1 propensity score matching in a single center of Korea. Among 1,448 patients who underwent index and surveillance colonoscopy, 210 matched pairs were analyzed. The 5-year cumulative occurrence of overall CRN after index colonoscopy was higher in the significant alcohol consumption group (defined as alcohol consumption more than 30 g/day in men and 20 g/day in women) (vs. without significant alcohol consumption group) (40% *vs*. 27.6%, *p* = 0.004). Significant alcohol consumption increased the development of overall CRN (adjusted hazard ratio [aHR]: 1.86, 95% confidence interval [CI]: 1.28–2.70, *p* = 0.001) at surveillance colonoscopy. However, this effect was not valid on the development of advanced CRN. In subgroup analysis considering the risk classification of index colonoscopy, significant alcohol consumption increased the overall CRN development at surveillance colonoscopy in the normal group (patients with no detected adenoma in the index colonoscopy) (aHR: 1.90, 95% CI: 1.16–3.13, *p* = 0.01). Alcohol consumption habits should be considered in optimizing time intervals of surveillance colonoscopy.

## Introduction

The role of surveillance colonoscopy has been well-known to prevent the occurrence of metachronous colorectal neoplasm (CRN) and colorectal cancer (CRC) in subjects with CRN at index colonoscopy^1,2^. The current recommendations for post-polypectomy surveillance colonoscopy suggested the 3–10 year interval of surveillance colonoscopy according to the presence of high risk findings (adenomas with villous histology or high-grade dysplasia, adenomas ≥10 mm in size, or ≥3 adenomas, serrated polyp ≥10 mm or dysplasia) at index colonoscopy^3,4^. However, the previous observational studies that were based on these guidelines mainly focused on the features of the polyps at index colonoscopy and rarely considered the patient-related risk factors of CRN^5–7^. Although a recent prospective study showed that metabolic syndrome influenced the development of CRN at surveillance colonoscopy^8^, the influence of personalized risk factors other than the findings of index colonoscopy on the occurrence of CRN at the time of surveillance colonoscopy has not been thoroughly elucidated to date.

Alcoholic beverages are well-known risk factors of multiple malignancies including head and neck-, breast-, prostate-, and gastrointestinal cancer^9,10^. In terms of CRC, two previous comprehensive meta-analyses showed that alcohol intake increased the risk of CRC by approximately 1.5-fold^11,12^. On the basis of the well-recognized adenoma-carcinoma sequence, several studies were conducted to determine the impact of alcohol consumption on the development of CRN, but these studies showed inconsistent results and have not been able to clearly identify the clinical association between alcohol consumption and CRN^13–15^. Recent meta-analysis showed that increased intake of 25 g per day of alcohol consumption was related to an increased risk of colorectal adenomas. However, there was considerable heterogeneity between studies not explained by study design, sex, geographic location, publication year, site or size of the lesions, type of adenoma, number of cases, endoscopic assessment, or adjustment for main confounders^16^. In addition, most studies assessed the association between alcohol consumption and the occurrences of CRN at the same time points. Therefore, the effect of alcohol consumption at the time of surveillance colonoscopy has not been investigated, and alcohol consumption was not included in the risk factors for the determination of the time interval of surveillance colonoscopy. This study aimed to determine if alcohol consumption is associated with the development of CRN at the time of surveillance colonoscopy.

## Materials and Methods

### Study population

A total of 4,578 subjects who underwent index colonoscopy between January 2009 and December 2013 at a single hospital were retrospectively identified. The short-term follow-up examination (within a year) due to poor bowel preparation or the incomplete resection of the noted polyp was identified from the chart review and excluded. Among these subjects, we selected 1,887 who received surveillance colonoscopy at least once up to December 2016. Out of that group, 439 subjects were ruled out according to the exclusion criteria as follows; 1) a prior history of colorectal disease or surgery (n = 52); 2) inadequate colonoscopy examination (n = 48); 3) any colonoscopies within the previous 3 years of the index colonoscopy (n = 175); and 4) an insufficient medical records (missing variables ≥2, n = 164). Finally, the remaining 1,448 subjects were enrolled in this study prior to the propensity score matching (PSM) analysis (Supplementary Fig. 1). This study was approved by the institutional review boards of Hallym University Chuncheon Sacred Heart Hospital (number: 2017–79) and performed in accordance with the Declaration of Helsinki. Informed consent was exempted due to retrospective format of this study from institutional review board of Chuncheon Sacred Heart Hospital and patients were not required to give informed consent to the study because the analysis used anonymous clinical data that were obtained after each patient agreed to treatment by written consent.

### Definitions and clinical outcomes

In this study, all colonoscopies were conducted by experienced endoscopists with fulfillment of cecal intubation and adequate withdrawal time (≥6 minutes). The details of colonoscopic procedures have been described in previously^17^. The overall CRN was defined as colorectal cancer or any adenoma, and advanced CRN was defined as invasive cancer or advanced adenoma (adenoma with villous components, high-grade dysplasia or size above 10 mm). We categorized the study population into normal (no adenoma), low- (1 or 2 adenomas less than 10 mm)-, and high- (advanced adenoma or more than 3 adenomas) risk groups based on the findings of the index colonoscopy to evaluate the association between the index colonoscopic findings and the development of overall CRN at surveillance colonoscopy. We defined the proximal colorectum as the ascending and transverse colon, and the distal colorectum as descending, sigmoid colon and rectum, respectively. We stipulated the significant alcohol consumption in the case of ethanol consumption more than 30 g/day in men and 20 g/day in women for recent years^18^.

The primary outcome was the difference in the cumulative rate of overall and advanced CRN occurrence at the time of surveillance colonoscopy between the alcohol consumption group and the without significant alcohol consumption group. The cumulative rate of overall CRN occurrence by risk categories at index colonoscopy was evaluated as a subgroup analysis to confirm the robustness of primary outcome. Additionally, we analyzed the risk factors for these outcomes, respectively.

### Data collection

We reviewed the clinical information from 1,448 subjects using electronic medical records. In terms of alcohol consumption, we collected more detailed data on the type of alcohol beverage, amount and frequency of alcohol intake for more than 2 years after the time of index colonoscopy using a self-reported recall questionnaire or interview by trained nurses. Interviews and questionnaires were conducted when each patient visited the hospital to reserve a date of colonoscopy (several days or weeks before colonoscopic procedures). The types of alcohol beverage that Koreans enjoy include Soju (Korean distilled spirits), Makgeolli (Korean traditional drink), beer, and wine. Alcohol consumption per day was calculated as the total amount of alcohol [drinking amount (ml) x alcohol content (%) x alcoholic density (0.8)] per week divided by 7 to estimate the average amount of alcohol intake per day. We identified the results of index and surveillances colonoscopies including the number, size, location, and pathological findings of biopsied or resected specimens. The interval between index and surveillance colonoscopy and the number of surveillance colonoscopies performed were also collected.

### Statistical analysis

PSM is a statistical method that attempts to estimate the effect of observed characteristics by accounting for the covariates that predict the effect of intervention and to balance the confounding variables to reduce the bias. This method allows for producing groups that are functionally randomized and can facilitate causal inferences from observational data^19^.

Continuous and categorical variables are presented as the mean ± standard deviation, and number and percentage, respectively. We estimated the differences of baseline characteristics between the subjects with and without significant alcohol consumption using chi-square tests and student’s *t*-tests for categorical and continuous variables, respectively. Thereafter, PSM was performed based on the presence of significant alcohol consumption to adjust for potential confounding factors and to identify the effect of significant alcohol consumption on clinical outcomes. Among variables that can be obtained through medical record review, questionnaire, or interview, we matched age, sex, BMI, smoking habits, the presence of diabetes or hypertension, the use of aspirin or non-steroidal anti-inflammatory drugs (NSAIDs) or lipid lowering agents, and the family history of CRC to adjust potential confounding effects according to the differences in baseline characteristics between the patients with and without significant alcohol consumption group. After matching, the absolute standardized mean differences to diagnose the balance after matching were less than 0.1. In the PS matched cohort, the cumulative rate for the clinical outcomes was estimated using the Kaplan-Meier method. Additionally, a Cox proportional hazards analysis was performed to evaluate the independent risk factors of each clinical outcome. The time metric of the above statistics was based on the month. Censoring was defined as the occurrence of CRN. If CRN was detected both in the 1^ST^ and 2^nd^ follow-up colonoscopy, the time of 1^ST^ follow-up colonoscopy was censored. All the potential confounding variables were included in the univariate analysis and multivariate analysis. A *p* value < 0.05 (2-tailed) was considered to be statistically significant in this study. All of the analyses were performed using SPSS version 24.0. (SPSS Inc., Chicago, IL, USA).

## Results

### Baseline characteristics

The baseline characteristics of the enrolled subjects based on the presences of significant alcohol consumption are summarized in Table 1. Among 1,448 subjects, the number of subjects with significant alcohol consumption and without significant alcohol consumption were 211 and 1,237, respectively. In the unmatched cohort, the subjects with significant alcohol consumption were male predominant (92.9% *vs*. 48.5%, *p* < 0.001) and younger (50.7 ± 9.6 *vs*. 54.8 ± 10.9, *p* < 0.001), and there was a higher proportion of subjects with ever smoking habits (65.9% *vs*. 19.6%, *p* < 0.001) compared with the subjects without significant alcohol consumption. The other baseline characteristics including findings of the index colonoscopy, the distributions of risk category, location, and the number of CRN were comparable between the two groups (*p*s > 0.05). The most subjects in significant alcohol consumption group and without significant alcohol consumption group underwent one surveillance colonoscopy with about 48-month interval, and about 10% of subjects underwent more than two surveillance colonoscopies. The mean time interval between the index and first- or second surveillance colonoscopy and the mean number of surveillance colonoscopies were similar between two groups.

**Table 1:** Baseline characteristics at the time of index colonoscopy in the unmatched and matched cohorts.

	Unmatched cohort			Matched cohort		
	With significant alcohol consumption (n = 211)	Without Significant alcohol consumption (n = 1,237)	*p* value	With significant alcohol consumption (n = 210)	Without significant alcohol consumption (n = 210)	*p* value
Sex (Men)	196 (92.9%)	600 (48.5%)	<0.001	195 (92.9%)	197 (93.8%)	0.85
Age (Years)	50.7 ± 9.6	54.8 ± 10.9	<0.001	50.7 ± 9.6	50.8 ± 10.2	0.89
BMI (kg/m^2^)	24.7 ± 3.6	24.5 ± 3.3	0.48	24.7 ± 3.5	24.6 ± 3.3	0.85
Ever- smoker	139 (65.9%)	242 (19.6%)	<0.001	138 (65.7%)	130 (61.9%)	0.48
Family history of CRC	6 (2.8%)	22 (1.8%)	0.29	6 (2.9%)	11 (5.2%)	0.32
Hypertension	69 (32.7)	382 (30.9)	0.63	68 (32.4%)	70 (33.3%)	0.92
Diabetes	32 (15.2%)	157 (12.7%)	0.32	31 (14.8%)	29 (13.8%)	0.89
Aspirin or NSAIDs use	27 (12.8%)	202 (16.3%)	0.22	27 (12.9%)	38 (18.1%)	0.18
Lipid lowering agent	29 (13.7%)	190 (15.4%)	0.60	29 (13.8%)	22 (10.5%)	0.37
**Index colonoscopy findings**						
Risk group			0.37			0.74
Normal	139 (65.9%)	868 (70.2%)		138 (65.7%)	144 (68.6%)	
Low risk	49 (23.2%)	262 (21.2%)		49 (23.3%)	47 (22.4%)	
High risk	23 (10.9%)	107 (8.6%)		23 (11.0%)	19 (9.0%)	
Number of CRN	72	369	0.27	72	66	>0.99
1 or 2 CRN	58 (80.6%)	313 (84.8%)		58 (80.6%)	53 (80.3%)	
3 or more CRNs	14 (19.4%)	56 (15.2%)		14 (19.4%)	13 (19.7%)	
Location of CRN	72	369	0.88	72	66	0.39
Proximal colorectum	30 (41.7%)	150 (40.7%)		30 (41.7%)	22 (33.3%)	
Distal colorectum	26 (36.1%)	126 (34.1%)		26 (36.1%)	25 (37.9%)	
Multiple sites	16 (22.2%)	93 (25.2%)		16 (22.2%)	19 (28.8%)	
**Surveillance colonoscopy**						
Number of surveillance colonoscopy						
Mean number of surveillance colonoscopy	1.1 ± 0.4	1.1 ± 0.4	0.43	1.1 ± 0.4	1.1 ± 0.3	0.50
More than 2 surveillance colonoscopies	27 (12.8%)	139 (11.2%)	0.55	26 (12.4%)	23 (11.0%)	0.69
**Interval from index to surveillance colonoscopy (months)**						
First surveillance colonoscopy	47.5 ± 14.5	48.3 ± 13.6	0.46	47.5 ± 14.5	49.9 ± 13.7	0.08
2^nd^ surveillance colonoscopy	62.3 ± 14.4	67.2 ± 12.1	0.06	62.2 ± 14.7	67.5 ± 11.4	0.17
Surveillance colonoscopy findings						
Overall CRN	70 (33.2%)	316 (25.5%)	0.02	70 (33.3%)	49 (23.3%)	0.03
Advanced CRN	7 (3.3%)	44 (3.6%)	> 0.99	7 (3.3%)	6 (2.9%)	>0.99

BMI, body mass index; CRC, colorectal cancer; NSAID, non-steroidal anti-inflammatory drugs; CRN, colorectal neoplasm. Significant alcohol consumption was defined as more than 20 g/day in women and 30 g/day in men. Risk category of index colonoscopy was defined as normal (no adenoma), low- (1 or 2 adenomas less than 10 mm)-, and high- (advanced adenoma or more than 3 adenomas) risk groups.

PSM analysis revealed 210 matched pairs of subjects classified according to significant alcohol consumption. After PSM analysis, between-group differences in sex, age, and smoking habits were no longer observed.

### Cumulative incidence and risk factors for overall and advanced CRN occurrence in the matched cohort

**Figure 1:**
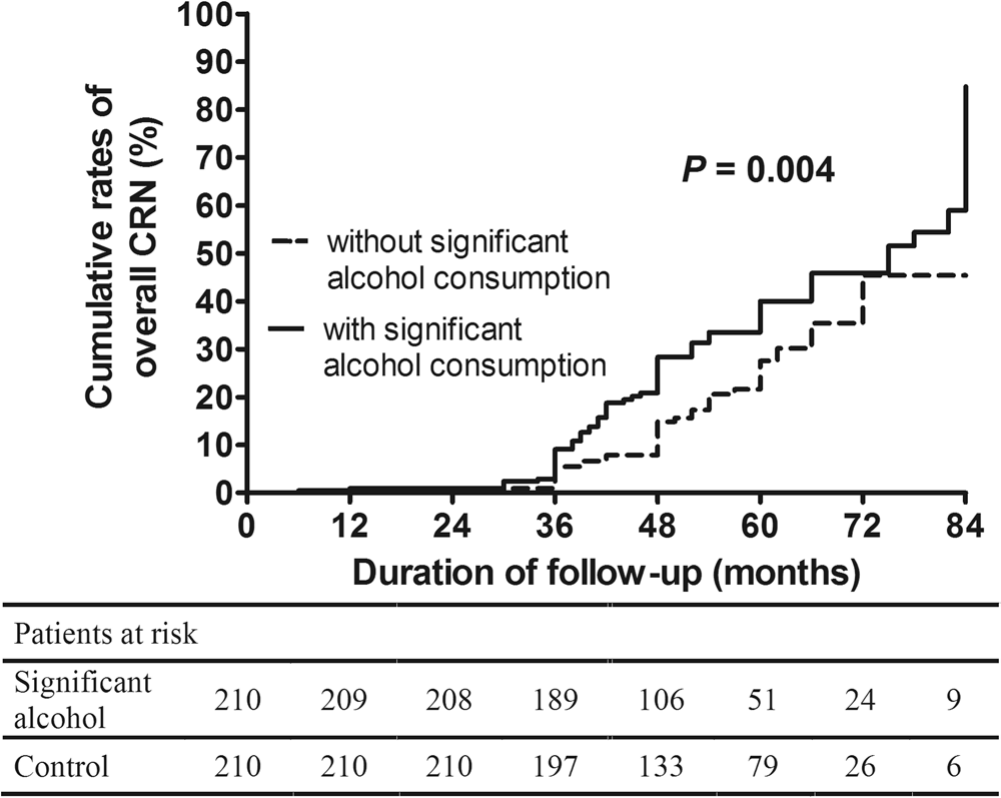
Cumulative rates of overall CRN occurrence in the significant alcohol consumption group and the without significant alcohol consumption group at the time of the surveillance colonoscopy. CRN, colorectal neoplasm.

Among 210 PS matched pairs, overall CRN was observed in 70 (33.3%) subjects in the significant alcohol consumption group and 49 (23.3%) subjects in the without significant alcohol consumption group during the surveillance period (*p* = 0.03). The cumulative rates of overall CRN occurrences at 3 (5.5% vs. 9.1%) and 5 (27.6% vs. 40.0%) years were significantly higher in the significant alcohol consumption group compared with the without significant alcohol consumption group (*p* = 0.004) (Fig. 1). In the Cox proportional hazard analysis, significant alcohol consumption (adjusted hazard ratio [aHR]: 1.86, 95% confidence interval [CI]: 1.28–2.70, *p* = 0.001) was associated with the risk of overall CRN occurrences at the time of surveillance colonoscopy; male (aHR: 2.73, 95% CI: 1.14–6.53, *p* = 0.02), BMI (aHR: 0.93, 95% CI: 0.88–0.98, *p* = 0.01), the presence of hypertension (aHR: 2.05, 95% CI: 1.38–3.04, *p* < 0.001), and high-risk findings of index colonoscopy (vs. normal group, aHR: 2.26, 95% CI: 1.38–3.73, *p* = 0.001) were also associated with this risk (Table 2).

**Table 2:** Cox proportional hazard analyses for the risk factors of colorectal neoplasm occurrence at the surveillance colonoscopy in the matched cohort.

Variables	Overall CRN occurrence (n = 119 from 420 matched cohort, 28.3%)				Advanced CRN occurrence (n = 13 from 420 matched cohort, 3.1%)			
	Univariate analysis		Multivariate analysis		Univariate analysis		Multivariate analysis	
	HR (95% CI)	*p* value	HR (95% CI)	*p* value	HR (95% CI)	*p* value	HR (95% CI)	*p* value
Sex (Men)	1.99 (0.87–4.58)	0.10	2.73 (1.14–6.53)	0.02	1.07 (0.14–8.25)	0.95	1.70 (0.20–14.57)	0.63
Age (Years)	1.02 (1.01–1.04)	0.007	1.01 (0.98–1.03)	0.64	1.08 (1.03–1.14)	0.002	1.08 (1.03–1.14)	0.003
BMI (kg/m^2^)	0.97 (0.91–1.02)	0.25	0.93 (0.88–0.98)	0.01	0.91 (0.77–1.09)	0.31	0.92 (0.77–1.11)	0.41
Current or past- Smoking	1.50 (1.01–2.25)	0.05	0.33 (0.87–2.03)	0.18	0.90 (0.30–2.77)	0.86	0.95 (0.27–3.39)	0.94
Family history of CRC	1.25 (0.51–3.07)	0.63	1.21 (0.47–3.14)	0.69	4.5 (1.00–20.34)	0.051	7.06 (1.28–39.09)	0.03
Hypertension	1.69 (1.17–2.44)	0.005	2.05 (1.38–3.04)	<0.001	2.73 (0.92–8.15)	0.07	1.80 (0.44–7.42)	0.41
Diabetes	1.61 (0.99–2.61)	0.05	1.46 (0.88–1.42)	0.14	1.29 (0.29–5.85)	0.74	1.24 (0.23–6.74)	0.80
Aspirin or NSAIDs use	1.04 (0.62–1.74)	0.89	0.67 (0.38–1.18)	0.16	2.71 (0.83–8.83)	0.10	1.58 (0.40–6.22)	0.52
Lipid lowering agent	1.07 (0.57–2.00)	0.84	0.75 (0.37–1.54)	0.43	0.75 (0.10–5.81)	0.78	0.49 (0.05–5.13)	0.56
Significant alcohol consumption	1.68 (1.16–2.43)	0.006	1.86 (1.28–2.70)	0.001	1.32 (0.44–3.94)	0.62	1.72 (0.53–5.53)	0.36
Index colonoscopy findings		0.005		0.005		0.001		0.007
Normal risk	Reference		Reference		Reference		Reference	
Low risk	1.41 (0.91–2.19)	0.12	1.29 (0.83–2.01)	0.26	1.28 (0.25–6.59)	0.77	0.69 (0.12–3.93)	0.68
High risk	2.24 (1.37–3.68)	0.001	2.26 (1.38–3.73)	0.001	8.77 (2.67–28.79)	<0.001	5.16 (1.51–17.61)	0.009

BMI, body mass index; CRC, colorectal cancer; NSAID, non-steroidal anti-inflammatory drugs; CRN, colorectal neoplasm; HR, hazard ratio; CI, confidence interval. In the multivariate analysis, sex, age, BMI, smoking, family history of CRC, hypertension, diabetes, aspirin, NSAIDs, or lipid lowering agent usage, proportion of patients with significant alcohol consumption, and index colonoscopy findings were controlled. Significant alcohol consumption was defined as more than 20 g/day in women and 30 g/day in men. Risk category of index colonoscopy was defined as normal (no adenoma), low- (1 or 2 adenomas less than 10 mm)-, and high- (advanced adenoma or more than 3 adenomas) risk groups.

The number of subjects with advanced CRN at surveillance colonoscopy were 7 (3.3%) and 6 (2.9%) in the significant alcohol consumption group and without significant alcohol consumption group, respectively (*p* > 0.99). These 13 advanced CRNs consisted of high-grade dysplasia (n = 2) and adenoma ≥1 cm (n = 12); invasive cancer or villous adenoma were not observed. The cumulative proportions of advanced CRN at 3 (0% vs. 1.0%) and 5 (5% vs. 4.5%) years were comparable between the two groups (*P* = 0.61). The risk factors for the development of advanced CRN were age (aHR: 1.08, 95% CI: 1.03–1.14, *p* = 0.003), family history of CRC (aHR: 7.06, 95% CI: 1.28–39.09, *p* = 0.03), and high-risk findings at the index colonoscopy (aHR: 5.16, 95% CI: 1.51–17.61, *p* = 0.009) in the Cox proportional hazard analysis (Table 2).

### Risk factors of overall CRN occurrence according to risk stratification based on the findings at index colonoscopy

**Figure 2:**
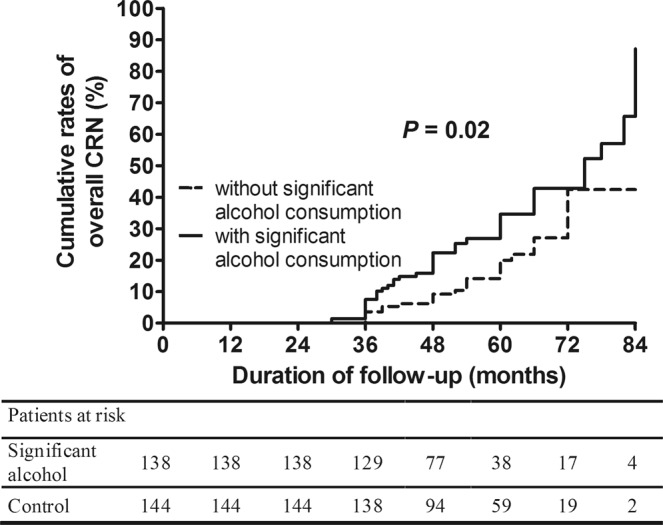
Cumulative rates of overall CRN occurrence in the significant alcohol consumption group and the without significant alcohol consumption group at the time of the surveillance colonoscopy according to risk stratification based on the findings at index colonoscopy (normal at index colonoscopy). CRN, colorectal neoplasm.

**Figure 3:**
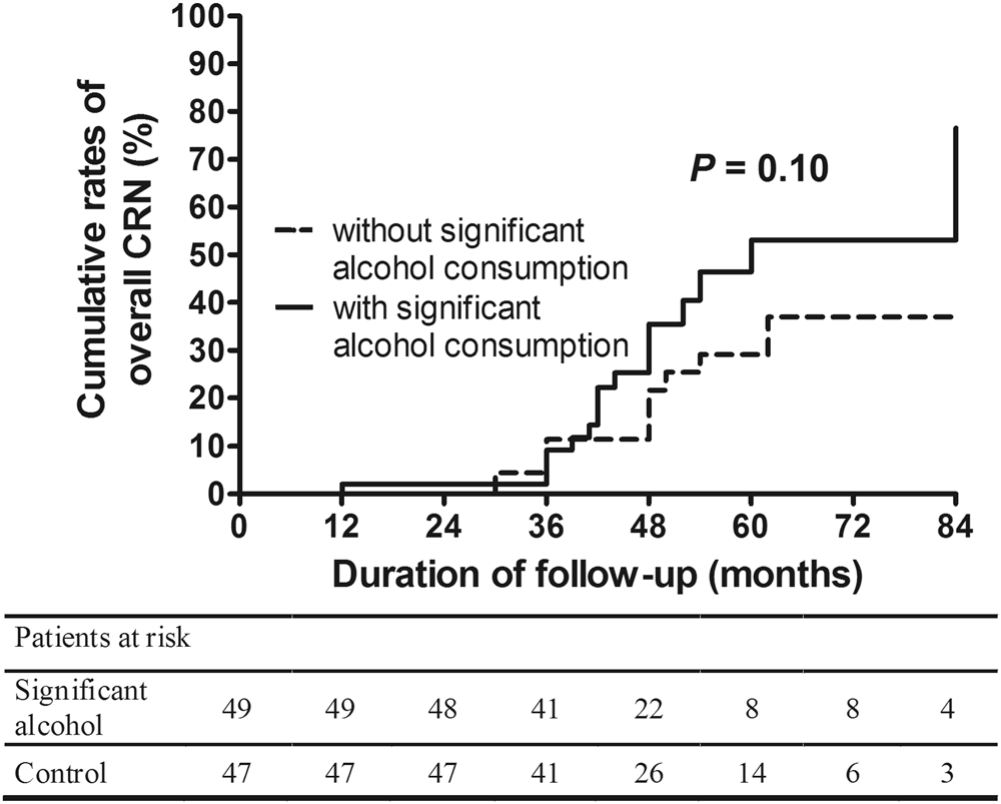
Cumulative rates of overall CRN occurrence in the significant alcohol consumption group and the without significant alcohol consumption group at the time of the surveillance colonoscopy according to risk stratification based on the findings at index colonoscopy (low-risk at index colonoscopy). CRN, colorectal neoplasm.

**Figure 4:**
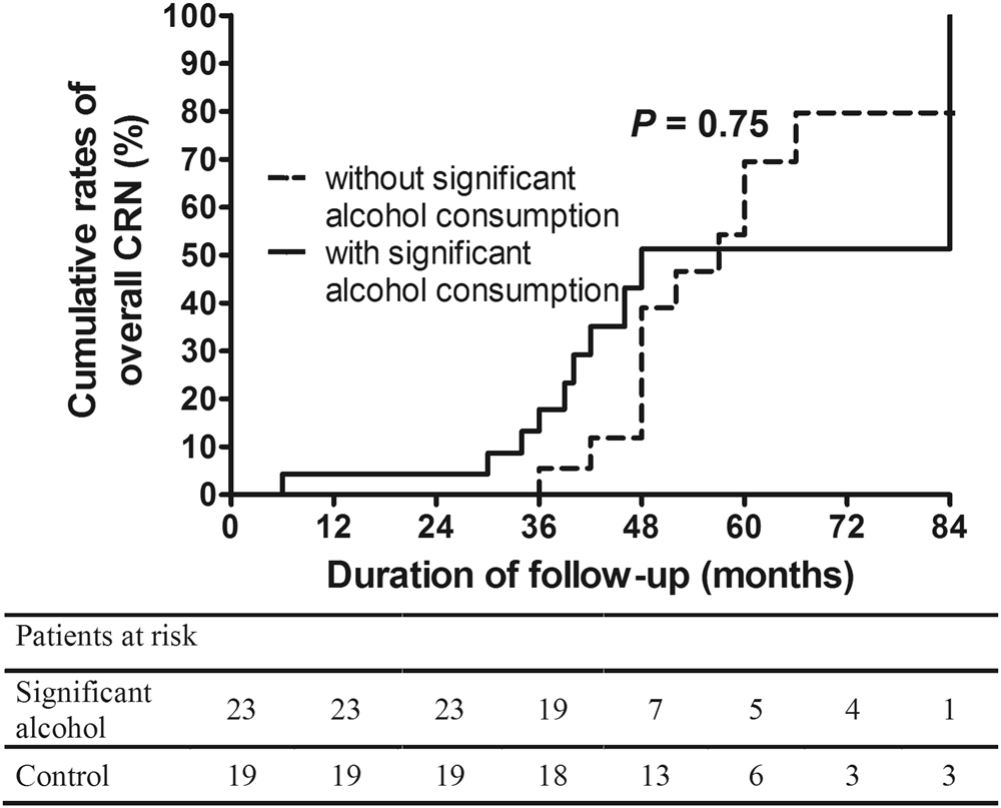
Cumulative rates of overall CRN occurrence in the significant alcohol consumption group and the without significant alcohol consumption group at the time of the surveillance colonoscopy according to risk stratification based on the findings at index colonoscopy (high-risk at index colonoscopy). CRN, colorectal neoplasm.

We analyzed the influence of significant alcohol consumption on the occurrence of overall CRN at surveillance colonoscopy according to classification by risk of the index colonoscopic findings. In the normal group, the 5-year cumulative rate of overall CRN occurrence was 34.6% in the significant alcohol consumption group, which was higher than that in the without significant alcohol consumption group (20.0%, *p* = 0.02) (Fig. 2) (aHR: 1.90, 95% CI: 1.16–3.13, *p* = 0.01) (Table 3). In the low-risk group, significant alcohol consumption also was associated with the development of overall CRN with marginal significance (aHR: 2.13, 95% CI: 0.98–4.62, *p* = 0.06) (Fig. 3) (Table 3). There was no statistical significance in the high-risk group, indicating a higher rate of overall CRN occurrence in subjects with significant alcohol consumption group (vs. without significant alcohol consumption group) (Fig. 4) (Table 3 & Supplementary Table 1).

**Table 3:** Subgroup analyses for the risk factors of overall CRN occurrence according to risk stratification based on the findings at index colonoscopy.

Risk category of index colonoscopy	Normal risk (n = 282, 67.1%) (overall CRN occurrence; n = 68 from 282 matched cohort, 24.1%)		Low risk (n = 96, 22.9%) (overall CRN occurrence; n = 29 from 96 matched cohort, 30.2%)		High risk (n = 42, 10%) (overall CRN occurrence; n = 22 from 42 matched cohort, 52.4%)	
Variables	HR (95% CI)	*p* value	HR (95% CI)	*p* value	HR (95% CI)	*p* value
Sex (male)	2.54 (0.88–7.31)	0.08	0.38 (0.05–3.18)	0.37	3.15 (0.34–29.51)	0.31
Age (Years)	1.01 (0.98–1.04)	0.42	1.00 (0.96–1.05)	0.94	0.98 (0.93–1.04)	0.52
BMI (kg/m^2^)	0.91 (0.84–0.99)	0.04	0.92 (0.82–1.02)	0.12	0.96 (0.82–1.11)	0.55
Current or ex- Smoking	1.03 (0.60–1.78)	0.92	1.59 (0.58–4.34)	0.37	2.12 (0.77–5.82)	0.14
Family history of CRC	1.78 (0.43–7.38)	0.43	1.30 (0.28–5.92)	0.55	0.47 (0.04–5.06)	0.53
Hypertension	1.67 (0.99–2.81)	0.05	2.13 (1.00–4.55)	0.05	2.05 (0.82–5.16)	0.13
Diabetes	1.41 (0.74–2.67)	0.30	1.81 (0.64–5.11)	0.26	0.93 (0.14–6.37)	0.95
Aspirin or NSAIDs use	0.61 (0.26–1.43)	0.25	1.42 (0.41–4.99)	0.58	0.68 (0.22–2.11)	0.51
Lipid lowering agent	0.66 (0.22–1.97)	0.46	0.67 (0.15–3.11)	0.61	0.40 (0.09–1.70)	0.22
Significant alcohol consumption	1.90 (1.16–3.13)	0.01	2.13 (0.98–4.62)	0.06	1.68 (0.66–4.26)	0.28

CRN, colorectal neoplasm; n, number; HR, hazard ratio; CI, confidence interval; BMI, body mass index; CRC, colorectal cancer; NSAID, non-steroidal anti-inflammatory drugs. In the multivariate analysis, sex, age, BMI, smoking, family history of CRC, hypertension, diabetes, aspirin, NSAIDs, or lipid lowering agent usage, and proportion of patients with significant alcohol consumption were controlled. Significant alcohol consumption was defined as more than 20 g/day in women and 30 g/day in men. Risk category of index colonoscopy was defined as normal (no adenoma), low- (1 or 2 adenomas less than 10 mm)-, and high- (advanced adenoma or more than 3 adenomas) risk groups.

### Risk factors of overall CRN occurrence at surveillance colonoscopy according to colorectal anatomic location

Of 119 subjects with overall CRNs found at surveillance colonoscopies, 49 and 51 had CRNs only in proximal and distal colorectum, respectively. Nineteen had CRNs in multiple anatomic sites. The 5-year cumulative rate of overall CRN occurrence at the distal colorectum was higher in the significant alcohol consumption group than in the without significant alcohol consumption group (19.1% *vs*. 10.0%, *p* = 0.01), while the 5-year cumulative rates of overall CRNs occurrence at the proximal colorectum (14.4% *vs*. 15.2%, *p* = 0.74) and multiple anatomic sites (9.4% *vs*. 4.1%, *p* = 0.07) were comparable between the 2 groups.

**Figure 5:**
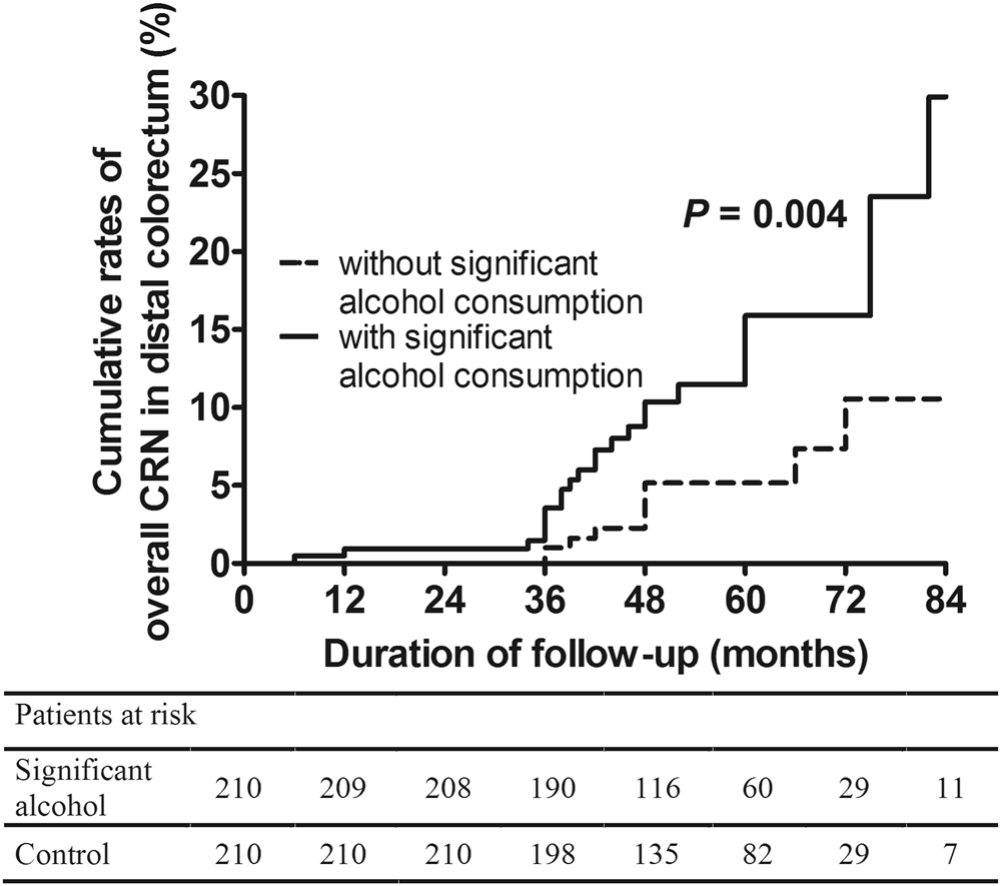
Cumulative rates of overall CRN occurrence at the distal colorectum in the significant alcohol consumption group and the without significant alcohol consumption group at the time of the surveillance colonoscopy. CRN, colorectal neoplasm.

Significant alcohol consumption was a risk factor for CRN occurrence at the distal colorectum (aHR: 2.01, 95% CI: 1.10–3.66, *p* = 0.02) together with ever-smoking (aHR: 2.68, 95% CI: 1.28–5.60, *p* = 0.009) and high-risk findings at the index colonoscopy (vs. the normal group, aHR: 2.55, 95% CI: 1.22–5.33, *p* = 0.01) (Supplementary Table 2) (Fig. 5). A statistical association between significant alcohol consumption and the occurrence of overall CRN in the proximal colorectum or multiple anatomic sites was not observed (Supplementary Tables 2, 3).

### Association between significant alcohol consumption and multiplicity of CRN occurrence at surveillance colonoscopy

Twelve (5.7%) and 5 (2.4%) subjects had 3 or more CRNs at the time of surveillance colonoscopy in the significant alcohol consumption group and the without significant alcohol consumption group, respectively (*p* = 0.14). Because there were no women among the subjects with CRNs ≥3 at the time of surveillance colonoscopy, we performed univariate and multivariate analysis only in males. Significant alcohol consumption appeared to increase the risk of multiple CRNs (≥3) occurrence at surveillance colonoscopy, although the result did not reach statistical significance (aHR: 2.60, 95% CI: 0.92–7.41, *p* = 0.07) (Supplementary Table 4).

## Discussion

To identify the potential risk factors of CRN occurrence at surveillance colonoscopy, we analyzed the influence of significant alcohol consumption on CRN development together with the findings of index colonoscopy. Our retrospective study with a well-balanced matched-pair design demonstrated that significant alcohol consumption doubled the risk of the overall CRN occurrence at surveillance colonoscopy, and this association was confined to the subjects with normal or low-risk findings at index colonoscopy. In addition, alcohol consumption was significantly associated with the development of overall CRN in the distal colorectum. Finally, significant alcohol consumption tended to show an increased risk of multiple CRNs (≥3) in males, although this result showed marginal significance. However, the association between significant alcohol consumption and the occurrence of advanced CRN at surveillance colonoscopy was not demonstrated in this study.

Several previous studies reported conflicting results about the relationship between alcohol consumption and CRN development. Jung *et al*. reported that alcohol consumption was not associated with CRN occurrence in a retrospective study of a large-scaled population^13^, whereas Zhu *et al*. showed that alcohol consumption increased the risk of CRNs by 17%, and the subjects with more than 50 g/day of alcohol consumption showed a significant association in a dose-response manner^20^. Similarly, a recent meta-analysis reported that alcohol consumption of more than 25 g/day increased the risk of CRN occurrence for both men and women^16^. These reports are in agreement with our findings. In our study, we defined significant alcohol consumption as more than 20 g/day in women and 30 g/day in men, and significant alcohol consumption increased the risk of overall CRN development.

In order to look at the risk of developing disease outcome during the follow-up period, the cohort members should not have prevalent outcome at baseline. The outcome of interest in this study was the CRN development. However, many patients had adenomas at baseline. For those patients, the outcome would be CRN recurrence and the risk of CRN recurrence would be different from that of CRN development among patients without prevalent adenomas at baseline. To compensate this issue, we performed separate analysis of patients with baseline no adenoma (normal group) and this also showed consistent result of significant alcohol consumption on the CRN development.

Because most previous studies excluded subjects with prior colonoscopy or subjects underwent colonoscopic polypectomy, studies that evaluated the time-dependent risk of alcohol consumption on CRN occurrence at surveillance colonoscopy, considered together with the risk categories of index colonoscopy, are rare. In this aspect, this study is the first to determine the effect of significant alcohol consumption after adjusting for the baseline results of index colonoscopic findings. Because there could be a concern about the subjects with more than 2 surveillance colonoscopies have possibility to be detected more CRNs, especially small CRNs, we performed additional analysis restricted to the first surveillance colonoscopy findings, and the effect of significant alcohol consumption on the overall CRN was unchanged (Supplementary Table 5). Also, because the interval and numbers of surveillance colonoscopies were similar between significant alcohol consumption group and without significant alcohol consumption group, multiple surveillance colonoscopies did not seem to affect the influence of significant alcohol consumption on the overall CRN development. Our study found that approximately 35% of subjects in the significant alcohol user in the normal group at index colonoscopy had overall CRN occurrence after 5 years of the index colonoscopy, and significant alcohol consumption increased the risk of more than 3 CRNs occurrence at surveillance colonoscopy in males, although this trend did not reach significance. The Recent post-polypectomy surveillance guidelines recommended 10-year surveillance intervals in patients with negative findings (normal) at the index colonoscopy^3^. However, Chu *et al*. reported that metabolic syndrome increased the risk of advanced CRN occurrence at surveillance colonoscopy in patients with negative findings at index colonoscopy and suggested a tailored approach for surveillance colonoscopy^8^. Given these findings, we could optimize surveillance intervals with regard to patient-related risk factors, especially in patients with negative findings at index colonoscopy. Additionally, because alcohol consumption is a modifiable risk factor for the recurrence of CRN, unlikely previous colonoscopic findings, we could suggest reducing or abstaining from alcohol to subjects with significant alcohol consumption to prevent recurrences of CRN.

Our study found that significant alcohol consumption doubled the risk of overall CRN at the distal colorectum. In contrast, there was no relationship between significant alcohol consumption and overall CRN at the proximal colorectum. Several previous studies that analyzed the relationship between significant alcohol consumption and adenomas at different sites of the colorectum showed inconsistent results. Ben *et al*. observed that alcohol consumption was associated with CRN occurrence only in the colon^16^, whereas Zhu and Baron *et al*. reported that alcohol consumption increased the risk of CRN occurrence in both the colon and rectum^20,21^. In a population-based case-control study, Toyomura *et al*. reported that alcohol consumption was associated with a modest increase in the risk of adenoma occurrence of the distal colon and rectal^22^, which was in keeping with our findings. The exact mechanism of alcohol consumption on CRN occurrence and possibly CRC occurrence is not fully established, although several possible mechanisms have been suggested. First, alcohol consumption results in folic acid deficiency, mainly due to folate malabsorption or preventing the enzyme for folic acid synthesis^23^. Second, intestinal microflora produces a considerable amount of acetaldehyde by oxidizing ethanol using alcohol dehydrogenase in the colorectum^24^. Acetaldehyde is thought to be a potential candidate for the carcinogenic effects of alcohol consumption^9^. Finally, alcohol consumption reduces immune surveillance, impairs DNA repairs, changes bile acids elements, and induces carcinogenic effects by the expression of cytochrome P-450 enzymes^25^. Further investigation will be important to evaluate to determine if the mechanism of carcinogenesis by alcohol consumption had different influences on CRN development depending on the anatomic sites.

In multivariate analysis of risk factors for overall CRN development, BMI was inversely related to the risk of CRN occurrences, which appears to be in conflict with the well-known concept that obesity increases the risk of CRN. Although exact reason cannot be investigated in our study, possible explanation is that BMI does not reflect the abdominal adiposity, which is more important for the development of CRN. Although BMI has been used as an indicator of obesity, previous studies have shown that the abdominal obesity, such as waist circumference is more associated with the development of CRN^26,27^. Also, most patients in our study were men with normal BMI, and it might be inappropriate to investigate the association of BMI with the development of CRN, although it was well-balanced matched cohort. Further studies with data on waist circumferences or lean body mass could reveal the association between abdominal obesity and CRN development.

The retrospective nature of this study leads to several limitations. First, there was a possibility of imperfectness of clinical information, including drinking pattern, lifetime alcohol consumption, and the type of alcohol beverage. Second, we could not investigate the habitual changes in alcohol consumption during the follow-up period. Third, selection bias could be observed in this study. Because the number of female subjects with significant alcohol consumption was insufficient, caution is needed to interpret the findings of our study, especially the hazard ratios of advanced CRN. Excluding patients with an insufficient medical record could attribute this issue. Among the 4,578 patients who underwent index colonoscopy, only 1,887 patients who underwent surveillance colonoscopy at least once were enrolled in the study; therefore, some degree of selection bias was also likely present. Further study is needed with increasing follow-up period to register sufficient number of patients to compensate this issue. Fourth, the interval of surveillance examination was relatively short. Lack of reasonable government policy about surveillance interval or public fear for colon cancer due to media coverage is presumed as the main reasons. Fifth, although the impact of alcohol consumption on the surveillance colonoscopy was demonstrated in this study, there was no evidence of the impact on the index colonoscopic findings. The objective of this study was not to determine the effect of alcohol on the index colonoscopic findings, therefore another study with different inclusion criteria should be performed. Sixth, there could be limited statistical power due to loss of many patients in the cohort after PSM. The main result of this study was that the cumulative rate of overall CRN at 5 years after index colonoscopy was higher in the significant alcohol consumption group (vs. without significant alcohol consumption group) (40% vs. 27.6%). Assuming randomized controlled trial for the power analysis with α-error <0.05 and a β-error <0.2 for a 2-tailed significance test (anticipated rate: 40% vs. 27.6%), the calculated number of subjects required for the trial is 227 in each arm. Assuming α-error <0.05 and a β-error <0.25 for a 2-tailed significance test, the calculated number of subjects required for the trial is 201 in each arm, although this study was not a randomized trial.

Although the limitations above, PSM produced well-balanced matched cohort for the statistical analysis. Therefore, significant alcohol consumption was associated with the risk of overall CRN occurrence at surveillance colonoscopy in subjects with normal or low-risk findings at index colonoscopy. In addition, significant alcohol consumption was associated with overall CRN occurrence in the distal colorectum and with more than 3 CRNs occurrence at surveillance colonoscopy. The post-polypectomy surveillance interval should be modified considering personalized potential risk factors of CRN development, especially in subjects with normal or low-risk findings at index colonoscopy.

## Supplementary information

Supplementary tables and figure
