# Supplementary material for: Alcohol consumption is associated with the risk of developing colorectal neoplasia: Propensity score matching analysis
Source: Sci Rep. 2019 Jun 4;9:8253. doi: 10.1038/s41598-019-44719-w (PMC6547846; doi:10.1038/s41598-019-44719-w)
Supplement: Supplementary file 1 — Supplementary tables and figure [file 41598_2019_44719_MOESM1_ESM.pdf]

Alcohol consumption is associated with the risk of developing colorectal neoplasia: Propensity score matching analysis

Young Joo Yang[1], Chang Seok Bang[1,2], Jae Ho Choi[2], Jae Jun Lee[2,3], Suk Pyo Shin[1], Ki Tae Suk[1], Gwang Ho Baik[1] & Dong Joon Kim[1]

Supplementary Fig. 1 Flow chart of the study

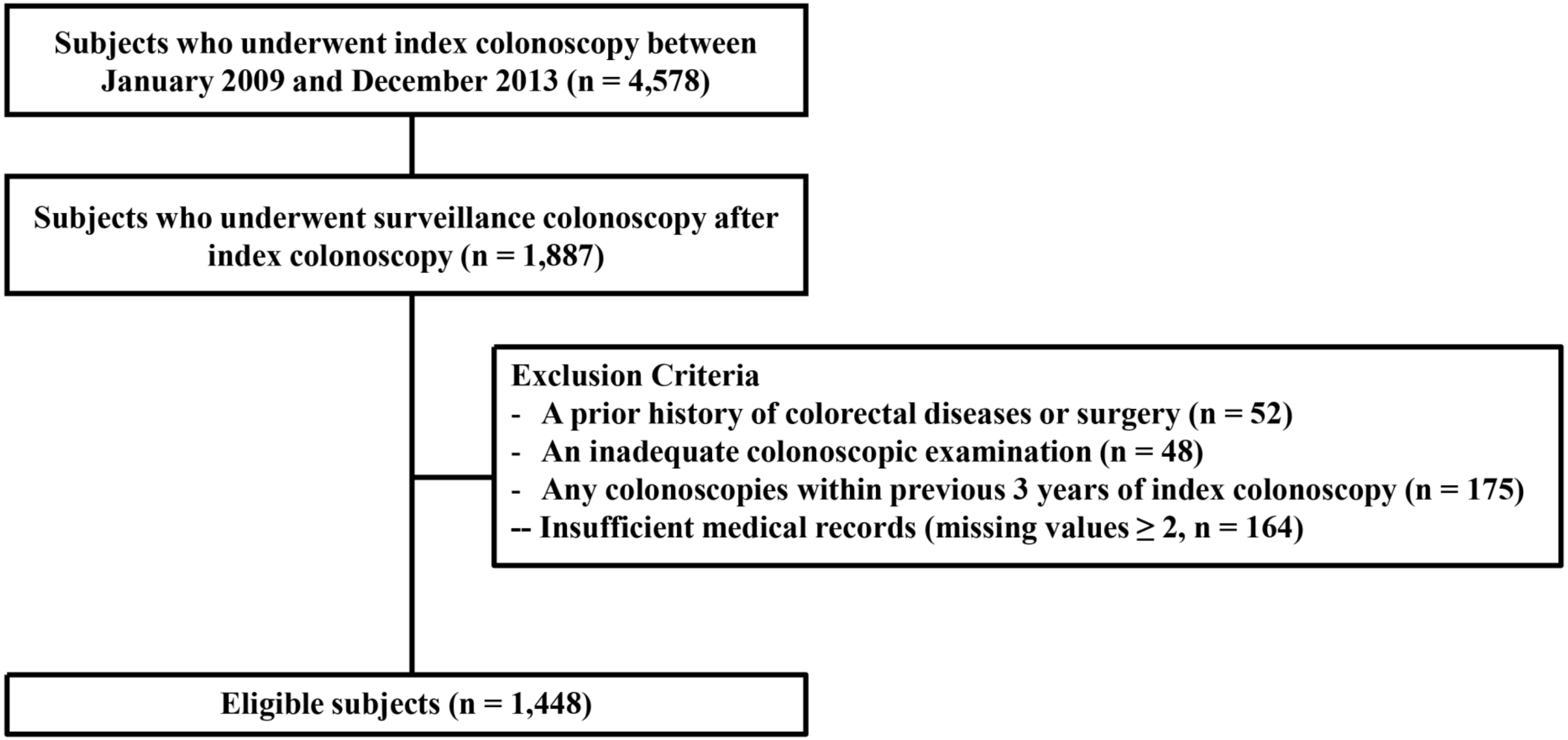

Supplementary Table 1 Univariate analysis for the risk factors of overall CRN occurrence according to risk stratification based on the findings at index colonoscopy

	**Normal**		**Low risk**		**High risk**	
	**HR (95% CI)**	***p* value**	**HR (95% CI)**	***p* value**	**HR (95% CI)**	***p* value**
**Sex (male)**	1.90 (0.69-5.24)	0.22	0.46 (0.06-3.38)	0.44	3.48 (0.45-27.14)	0.24
**Age**	1.03 (1.00-1.05)	0.04	1.02 (0.98-1.06)	0.35	1.02 (0.98-1.06)	0.36
**BMI (kg/m^2)**	0.95 (0.88-1.03)	0.23	0.96 (0.86-1.07)	0.47	0.97 (0.87-1.07)	0.52
**Current or ex- Smoking**	1.22 (0.73-2.03)	0.45	1.56 (0.63-3.83)	0.33	2.12 (0.77-5.82)	0.14
**Family history of CRC**	1.45 (0.36-5.95)	0.60	1.17 (0.28-4.98)	0.83	0.52 (0.07-3.92)	0.53
**Hypertension**	1.50 (0.90-2.49)	0.12	1.83 (0.88-3.83)	0.11	1.73 (0.71-4.19)	0.23
**Diabetes**	1.64 (0.88-3.08)	0.12	1.63 (0.61-4.34)	0.33	1.38 (0.38-4.93)	0.62
**Aspirin or NSAIDs use**	0.85 (0.39-1.88)	0.69	1.72 (0.59-5.00)	0.32	1.00 (0.36-2.76)	0.99
**Lipid lowering agent**	0.73 (0.26-2.02)	0.54	1.74 (0.60-5.08)	0.31	0.92 (0.27-3.19)	0.90
**Significant Alcohol**	1.78 (1.09-2.93)	0.02	1.85 (0.87-3.94)	0.11	1.15 (0.48-2.72)	0.76

BMI, body mass index; CRC, colorectal cancer; NSAID, non-steroidal anti-inflammatory drugs; CRN, colorectal neoplasm; HR, hazard ratio; CI, confidence interval.

Supplementary Table 2. Analyses for the risk factors of overall CRN occurrence according to colorectal anatomic location at the surveillance colonoscopy.

Anatomic location of CNR occurrence	Proximal colorectum (n = 49, 11.7%)					Distal colorectum (n = 51, 12.1%)				
Variables	n (n = 49)	Univariate analysis		Multivariate analysis		n (n = 51)	Univariate analysis		Multivariate analysis	
		HR (95% CI)	*p* value	HR (95% CI)	*p* value		HR (95% CI)	*p* value	HR (95% CI)	*p* value
**Sex (Men)**	46 (93.9%)	1.55 (0.48-5.06)	0.47	1.51 (0.45-5.06)	0.50	48 (94.1%)	2.55 (0.60-10.73)	0.20	2.56 (0.54-12.14)	0.24
**Age (Years)**	53.10 ± 10.39	1.03 (1.00-1.06)	0.03	1.02 (0.99-1.05)	0.21	52.24 ±10.50	1.02 (0.99-1.04)	0.30	0.99 (0.96-1.02)	0.57
**BMI (kg/m^2)**	24.64 ± 3.34	0.99 (0.91-1.08)	0.78	0.97 (0.89-1.06)	0.52	24.33± 3.73	0.95 (0.87-1.04)	0.28	0.92 (0.84-1.01)	0.09
**Current or past- Smoking**	32 (65.3%)	1.08 (0.60-1.95)	0.79	1.08 (0.58-2.03)	0.80	41 (80.4%)	2.61 (1.27-5.38)	0.009	2.68 (1.28-5.60)	0.009
**Family history of CRC**	3 (6.1%)	1.64 (0.51-5.29)	0.41	2.19(0.66-7.23)	0.20	2 (3.9%)	1.20 (0.29-4.94)	0.80	1.22 (0.27-5.47)	0.80
**Hypertension**	22 (44.9%)	1.99 (1.13-3.50)	0.02	2.51 (1.38-4.58)	0.003	21 (41.2%)	1.57 (0.89-2.78)	0.12	1.78 (0.95-3.34)	0.07
**Diabetes**	9 (18.4%)	1.78 (0.86-3.68)	0.12	1.94 (0.91-4.13)	0.09	11 (21.6%)	2.19 (1.11-4.31)	0.02	1.97 (0.97-4.02)	0.06
**Aspirin or NSAIDs use**	5 (10.2%)	0.70 (0.28-1.77)	0.45	0.33 (0.12-0.90)	0.03	10 (19.6%)	1.30 (0.63-2.70)	0.48	0.95 (0.41-2.19)	0.91
**Lipid lowering agent**	6 (12.2%)	1.40 (0.59-3.31)	0.45	1.16 (0.43-3.18)	0.77	4 (7.8%)	0.88 (0.32-2.48)	0.81	0.52 (0.16-1.67)	0.27

**Significant alcohol**	24 (49.0%)	1.10 (0.63-1.93)	0.74	1.05 (0.59-1.88)	0.87	33 (64.7%)	2.10 (1.16-3.77)	0.01	2.01 (1.10-3.66)	0.02
**Index colonoscopy findings**			0.67		0.94			0.06		0.04
**Normal risk**	31 (63.3%)	Reference				29 (56.9%)	Reference			
**Low risk**	12 (24.5%)	1.31 (0.67-2.55)	0.43	1.03 (0.52-2.07)	0.93	11 (21.6%)	1.10 (0.55-2.23)	0.78	1.01 (0.50-2.05)	0.97
**High risk**	6 (12.2%)	1.29 (0.54-3.10)	0.57	1.18 (0.47-2.96)	0.73	11 (21.6%)	2.40 (1.16-5.00)	0.02	2.55 (1.22-5.33)	0.01

BMI, body mass index; CRC, colorectal cancer; NSAID, non-steroidal anti-inflammatory drugs; CRN, colorectal neoplasm; HR, hazard ratio; CI, confidence interval.

In the multivariate analysis, sex, age, BMI, smoking, family history of CRC, hypertension, diabetes, aspirin, NSAIDs, or lipid lowering agent usage, proportion of patients with significant alcohol consumption, and index colonoscopy findings were controlled.

Supplementary Table 3 Analyses for the risk factors of overall CRN occurrence in multiple anatomic sites at the surveillance colonoscopy in matched patients

	Overall CRN occurrence in multiple anatomic sites (n = 19 from 420 matched cohort, 4.5%)				
**Variables**	n	**Univariate analysis**		**Multivariate analysis**	
		**HR (95% CI)**	***p* value**	**HR (95% CI)**	***p* value**
**Sex (male)**	18 (94.7%)	1.62 (0.22-12.13)	0.64	2.73 (0.31-23.90)	0.62
**Age**	53.05 ± 6.74	1.03 (0.99-1.08)	0.18	1.01 (0.96-1.07)	0.36
**BMI (kg/m^2)**	24.29 ± 5.52	0.95 (0.82-1.10)	0.49	0.94 (0.80-1.09)	0.41
**Current or ex- Smoking**	12 (63.2%)	0.91 (0.36-2.32)	0.84	0.57 (0.20-1.59)	0.28
**Family history of CRC**	0 (0%)	0.05 (0-1286.67)	0.56	0	0.99
**Hypertension**	6 (31.6%)	1.11 (0.42-2.94)	0.83	1.22 (0.37-4.06)	0.75
**Diabetes**	0 (0%)	0.04 (0-19.17)	0.31	0	0.98
**Aspirin or NSAIDs use**	3 (15.8%)	1.23 (0.36-4.23)	0.74	1.40 (0.34-5.68)	0.64
**Lipid lowering agent**	1 (5.3%)	0.55 (0.07-4.18)	0.57	0.80 (0.09-6.96)	0.84
**Significant alcohol**	13 (68.4%)	2.38 (0.9--6.28)	0.08	2.74 (0.98-7.68)	0.06
**Index colonoscopy findings**			0.05		0.06
**Normal risk**	8 (42.1%)	Reference		Reference	
**Low risk**	6 (31.6%)	2.24 (0.77-6.54)	0.14	2.07 (0.67-6.40)	0.20
**High risk**	5 (26.3%)	4.08 (1.32-12.63)	0.02	4.19 (1.25-14.07)	0.02

BMI, body mass index; CRC, colorectal cancer; NSAID, non-steroidal anti-inflammatory drugs; CRN, colorectal neoplasm; HR, hazard ratio; CI, confidence interval.

In the multivariate analysis, sex, age, BMI, smoking, family history of CRC, hypertension, diabetes, aspirin, NSAIDs, or lipid lowering agent usage, proportion of patients with significant alcohol consumption, and index colonoscopy findings were controlled.

Supplementary Table 4 Analyses for the risk factors of more than 3 CRNs occurrence at the surveillance colonoscopy in matched male patients

	More than 3 CRNs occurrence (n = 17 from 392 matched cohort, 4.3%)				
**Variables**	**n**	**Univariate analysis**		**Multivariate analysis**	
		**HR (95% CI)**	***p* value**	**HR (95% CI)**	***p* value**
**Age (Years)**	53.41 ± 6.85	1.03 (0.98-1.08)	0.21	1.01 (0.95-1.06)	0.87
**BMI (kg/m^2)**	24.98 ± 5.53	1.00 (0.87-1.15)	0.97	0.97 (0.84-1.13)	0.72
**Current or ex- Smoking**	14 (82.4%)	2.16 (0.62-7.51)	0.23	1.91 (0.54-6.72)	0.32
**Family history of CRC**	1 (5.9%)	1.59 (0.21-12.01)	0.65	1.23 (0.14-10.62)	0.85
**Hypertension**	7 (41.2%)	1.64 (0.62-4.32)	0.32	1.70 (0.63-4.60)	0.30
**Diabetes**	0 (0%)	0.04 (0-21.57)	0.32	0 (0)	0.98
**Aspirin or NSAIDs use**	2 (11.8%)	0.84 (0.19-3.70)	0.82	0.60 (0.13-2.84)	0.52
**Lipid lowering agent**	2 (11.8%)	1.16 (0.26-5.10)	0.85	0.67 (0.13-3.56)	0.64
**Significant alcohol**	12 (70.6%)	2.68 (0.94-7.62)	0.07	2.60 (0.92-7.41)	0.07
**Index colonoscopy findings**			<0.001		<0.001
**Normal risk**	5 (29.4%)	Reference		Reference	
**Low risk**	5(29.4%)	2.83 (0.81-9.84)	0.10	2.69 (0.77-9.38)	0.12
**High risk**	7 (41.2%)	9.79 (3.09-31.00)	<0.001	9.63 (3.05-30.46)	<0.001

BMI, body mass index; CRC, colorectal cancer; NSAID, non-steroidal anti-inflammatory drugs; CRN, colorectal neoplasm; HR, hazard ratio; CI, confidence interval.

In the multivariate analysis, age, BMI, smoking, family history of CRC, hypertension, diabetes, aspirin, NSAIDs, or lipid lowering agent usage, proportion of patients with significant alcohol consumption, and index colonoscopy findings were controlled.

Supplementary Table 5 Analyses for the risk factors of colorectal neoplasm occurrence at the first surveillance colonoscopy in matched patients

	Overall CRN occurrence (n = 110 from 420 matched cohort, 26.2%)					Advanced CRN occurrence (n = 10 from 420 matched cohort, 2.4%)				
**Variables**	n	**Univariate analysis**		**Multivariate analysis**		n	**Univariate analysis**		**Multivariate analysis**	
		**HR (95% CI)**	***p* value**	**HR (95% CI)**	***p* value**		**HR (95% CI)**	***p* value**	**HR (95% CI)**	***p* value**
**Sex (Men)**	104 (94.5%)	1.52 (0.67-3.49)	0.32	1.44 (0.59-3.55)	0.43	9 (90.0%)	0.70 (0.09-5.50)	0.73	1.02 (0.10-10.53)	0.99
**Age (Years)**	52.59 ± 9.72	1.30 (1.01-1.05)	0.002	1.01 (0.99-1.03)	0.27	58.30 ± 11.95	1.08 (1.02-1.14)	0.01	1.09 (1.03-1.15)	0.004
**BMI (kg/m^2)**	24.38 ± 3.95	0.96 (0.90-1.01)	0.12	0.93 (0.88-0.99)	0.02	23.35 ± 3.27	0.87 (0.71-1.06)	0.16	0.85 (0.67-1.07)	0.16
**Current or past- Smoking**	80 (72.7%)	1.50 (0.98-2.28)	0.06	1.46 (0.95-2.23)	0.08	6 (60.0%)	0.86 (0.24-3.04)	0.81	1.09 (0.37-4.43)	0.39
**Family history of CRC**	5 (4.5%)	1.38 (0.56-3.38)	0.49	1.34 (0.52-3.47)	0.54	2 (20.0%)	6.80 (1.44-32.06)	0.02	12.59 (2.19-72.55)	0.005
**Hypertension**	45 (40.9%)	1.75 (1.19-2.56)	0.005	2.14 (1.42-3.23)	<0.001	6 (60.0%)	3.46 (0.98-12.27)	0.06	2.34 (0.35-15.82)	0.38
**Diabetes**	18 (16.4%)	1.55 (0.93-2.59)	0.09	1.41 (0.83-2.40)	0.21	1 (10.0%)	0.79 (0.10-6.24)	0.82	0.51 (0.05-4.89)	0.56
**Aspirin or NSAIDs use**	16 (14.5%)	1.14 (0.67-1.94)	0.64	0.73 (0.41-1.30)	0.29	4 (40.0%)	4.07 (1.15-14.43)	0.03	2.26 (0.58-8.80)	0.24

**Lipid lowering agent**	11 (10.0%)	1.12 (0.60-2.11)	0.72	0.78 (0.38-1.63)	0.51	1 (10.0%)	0.98 (0.12-7.73)	0.98	0.77 (0.06-10.36)	0.84
**Significant alcohol**	67 (60.9%)	1.74 (1.18-2.56)	0.005	1.83 (1.24-2.71)	0.003	5 (50.0%)	1.18 (0.34-4.09)	0.79	1.65 (0.43-6.29)	0.46
**Index colonoscopy findings**			0.001		0.006			0.01		0.04
**Normal risk**	63 (57.3%)	Reference		Reference		5 (50.0%)	Reference			
**Low risk**	28 (25.5%)	1.56 (0.99-2.45)	0.05	1.42 (0.90-2.24)	0.13	1 (10.0%)	0.70 (0.08-6.02)	0.75	0.31 (0.03-2.99)	0.31
**High risk**	19 (17.3%)	2.49 (1.49-4.18)	0.001	2.30 (1.37-3.86)	0.002	4 (40.0%)	6.31 (1.69-23.57)	0.006	3.71 (0.97-14.14)	0.06

n, number; BMI, body mass index; CRC, colorectal cancer; NSAID, non-steroidal anti-inflammatory drugs; CRN, colorectal neoplasm; HR, hazard ratio; CI, confidence interval. In the multivariate analysis, sex, age, BMI, smoking, family history of CRC, hypertension, diabetes, aspirin, NSAIDs, or lipid lowering agent usage, proportion of patients with significant alcohol consumption, and index colonoscopy findings were controlled.
